# Structural Memory Effects in Gold–4,4′-Bipyridine–Gold Single-Molecule Nanowires

**DOI:** 10.1021/acs.jpclett.0c03765

**Published:** 2021-02-11

**Authors:** A. Magyarkuti, Z. Balogh, G. Mezei, A. Halbritter

**Affiliations:** † Department of Physics, Budapest University of Technology and Economics, Budafoki ut 8, 1111 Budapest, Hungary; ‡ MTA-BME Condensed Matter Research Group, Budafoki ut 8, 1111 Budapest, Hungary

## Abstract

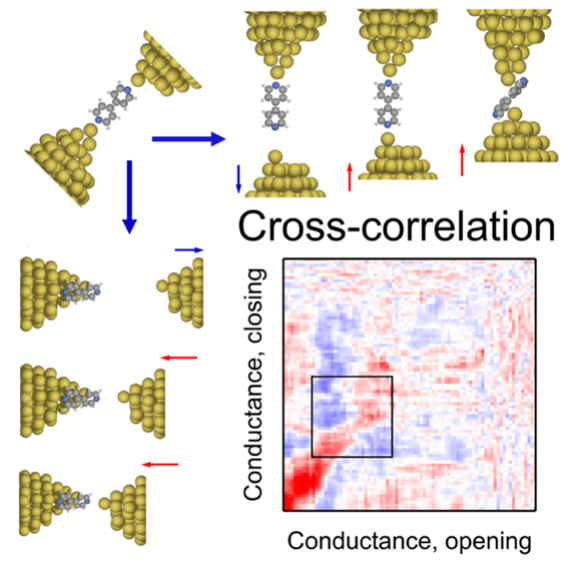

We study the vulnerability of single-molecule nanowires against a temporary disconnection of the junction. To this end, we compare the room and low-temperature junction formation trajectories along the opening and closing of gold–4,4′-bipyridine–gold single-molecule nanowires. In the low-temperature measurements, the cross-correlations between the opening and subsequent closing conductance traces demonstrate a strong structural memory effect: around half of the molecular opening traces exhibit similar, statistically dependent molecular features as the junction is closed again. This means that the junction stays rigid and the molecule remains protruding from one electrode even after the rupture of the junction, and therefore, the same single-molecule junction can be reestablished if the electrodes are closed again. In the room-temperature measurements, however, weak opening–closing correlations are found, indicating a significant rearrangement of the junction after the rupture and the related loss of structural memory effects.

Single-molecule electronics promises electronic building blocks with the ultimate smallest active volume combined with a rich chemical complexity.^1−4^ Such small feature sizes, however, are inevitably accompanied by a severe vulnerability: a sub-Ångström displacement of the electrodes may already induce a conformational change of the molecular junction.^5−7^ In this paper, we investigate the self-protection of single-molecule junctions against environmental perturbations by studying the structural memory effects of gold–4,4′-bipyridine (BP)–gold single-molecule structures.

BP molecules can attach to gold electrodes in two different binding geometries, resulting in double-step molecular plateaus.^6,8−17^ Similar features were observed with other pyridine-linked molecules as well.^18,19^ At a smaller electrode separation, the molecule binds on the side of the metallic junction, such that both the nitrogen linker and the aromatic ring are electronically coupled to the metal electrode (HighG configuration). Upon further pulling, the molecule slides to the apex and only the linkers couple to the electrodes, yielding a decreased junction conductance (LowG configuration). Here, we study the stability of these molecular arrangements investigating the two possible scenarios in Figure 1A,B. In the first case (Figure 1A), the junction stays rigid and the molecule remains protruding from one electrode even after the rupture, and therefore, the same single-molecule junction can be reestablished when the electrodes are closed again. This means that the molecular junction is not irreversibly lost upon a temporary disconnection. Alternatively, the junction may significantly rearrange after its rupture: for instance the molecule flips to the side of the electrodes (Figure 1B), or even the metallic apexes are reshaped. In this case, a temporary disconnection yields a loss of the molecular junction. To address this question, we investigate the correlations between the opening and closing junction formation trajectories in mechanically controllable break junction (MCBJ) measurements both at room temperature and at cryogenic circumstances (*T* = 4.2 K).

**Figure 1:**
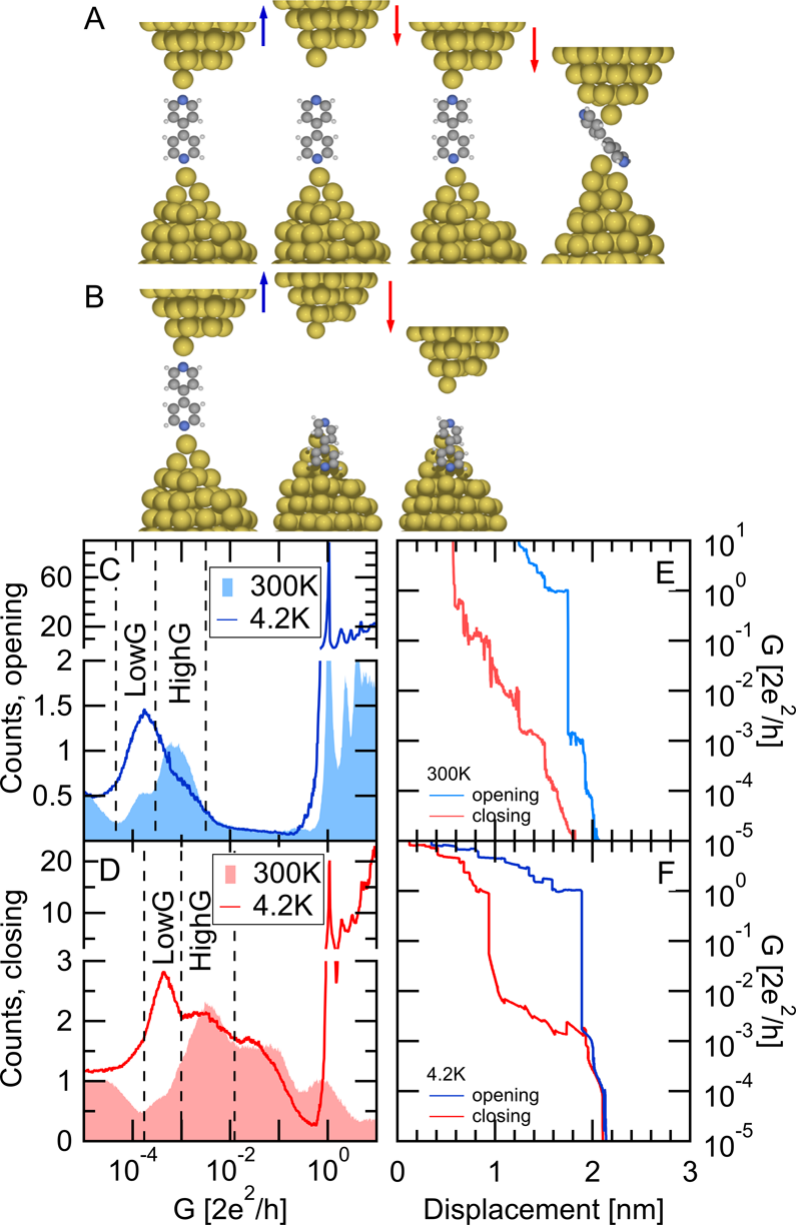
Illustration of two possible trajectories along the opening and subsequent closing of a single-molecule nanowire: a stable, rigid junction (A) or the loss of the molecular junction (B). Conductance histograms of room-temperature (area graphs) and low-temperature (lines) measurements during the opening (C) and closing (D) of the junction. The HighG and LowG conductance regions are indicated by dashed lines. In the closing histogram, these regions shift to higher conductances due to the less stretched nature of the molecular configurations. (E, F) Sample opening (blue)–closing (red) trace pairs at room temperature (E) and at low temperature (F). The displacement axis is calibrated by clean gold junctions before dosing the molecules. At low temperature, the calibration is based on the period of the peaks in the plateaus’ length histograms,^20,21^ whereas at room temperature, the MCBJ displacement ratio is set to achieve the same average slope of the tunneling traces as in calibrated scanning tunneling microscope (STM) break junction measurements. Afterward the molecules were introduced to the junction using an in situ evaporation technique.^17,22^ The room/low-temperature data include 5000/5500 conductance traces.

First, we compare the conductance histograms of the low- and room-temperature measurements. In accordance with earlier studies,^6^ the room-temperature opening traces yield a double-peak feature (area graph in Figure 1C). This corresponds to the HighG and LowG configurations of the BP molecule with conductances of ≈10^–3^*G*_0_ and ≈3 × 10^–4^*G*_0_, where *G*_0_ = 2*e*^2^/*h* ≈ (12.9 kΩ)^−1^ is the conductance quantum unit. On the other hand, the low-temperature measurements exhibit one dominant peak at the LowG conductance and only a shoulder is observed around the HighG conductance (blue line in Figure 1C). The agreement of the peak (and shoulder) positions suggests that similar junction geometries are sampled in the two measurements,^16^ but with different relative weights. This is related to the frequent formation of monatomic chains at low-temperature.^20^ Once the chain breaks, a wider gap is established which cannot accommodate the HighG configuration. This means that the HighG plateau is bypassed, and the corresponding histogram peak is suppressed.^16^

Next, we examine the closing histograms (Figure 1D). On a closing trace, first, a similar conductance plateau is observed as on the opening trace, but afterward, the conductance is increasing along an extended displacement range exceeding the conductance of the conventional LowG and HighG configurations but not yet reaching the conductance quantum unit (see the room- and low-temperature sample opening–closing trace pairs in Figure 1E,F). In the latter region, the tunneling leakage current between the electrodes may play an important role,^23^ and the molecule may slide between the junctions, as illustrated on the right cartoon of Figure 1A.

The low-temperature closing histogram (red line in Figure 1D) exhibits a peak somewhat above the conductance of the LowG configuration along the opening process, and a shoulder somewhat above the conductance of the opening HighG configuration. We argue that these are related to less stretched LowG and HighG configurations (see the dashed lines illustrating these two conductance ranges). We attribute the conductance difference between the opening and closing peak positions to the sampling of more stretched, and therefore less conductive configurations along the opening process (see the Supporting Information for a further analysis of this feature). The coexistence of the LowG peak in the opening and closing histogram indicates that the LowG configuration can be recovered as the junction is closed again in a low-temperature environment.

The room-temperature closing histogram (area graph in Figure 1D) exhibits a clear peak in the region of the less stretched HighG configuration, but in the less stretched LowG region only a very weak shoulder is observed. This indicates that even though the junction usually breaks from the LowG arrangement, this configuration is mostly lost after rupture; instead, the molecule rearranges such that only the tilted HighG configuration is available when the electrodes are closed again. As a further important remark, the peak at the conductance quantum unit is extremely weak compared either to the room-temperature opening histogram, or even to the low-temperature closing histogram. This indicates that the metallic apexes may flatten due to the strong surface diffusion of gold at room temperature,^24−27^ and therefore, immediately a larger area junction is formed when the metallic electrodes touch.

The two-dimensional conductance histograms (see Supporting Information) confirm that molecular plateaus extend to longer displacement during the closing of the junction. Furthermore, these two-dimensional histograms reveal that at low temperature, a significant number of traces are measured without molecular signatures; i.e., tunneling traces are mixed with molecular ones in both the opening and the closing direction. In contrast, the room-temperature measurements do not indicate significant number of tunneling traces in either direction, thus almost all opening and closing conductance traces exhibit molecular plateaus.

To investigate the relation between the different binding configurations, as well as the structural memory effects of molecular junctions, we apply the correlation analysis techniques, as introduced in refs (22 and 28−30), Figure 2 demonstrates the low- and room-temperature opening–opening and opening–closing correlation plots (Figure 2B,C,F,G with more details on the calculation of the correlation matrices in the figure caption), and the corresponding reference conductance histograms (Figure 2A,D,E,H). The autocorrelation plot of the opening traces measured at room temperature (Figure 2B) exhibits multiple correlated conductance regions in addition to the evident perfect correlation at the diagonal. Here we focus on the correlations between the two molecular configurations (see the enframed region and the dashed lines indicating the same borders of the molecular configurations as in Figure 1C,D). The off-diagonal blocks of the enframed region, i.e., the correlations between the HighG and LowG configurations, exhibit pronounced negative values. Such an anticorrelation may originate either from the *existence* or from the *length* of the plateaus. In the first case, either the one or the other plateau is observed but not both. In the latter case, both plateaus are observed, but their lengths are anticorrelated: a shorter than average plateau in one configuration is accompanied by a longer than average plateau in the other configuration. In case of the two BP binding configurations, the latter type of anticorrelation was reported in ref (28).

**Figure 2:**
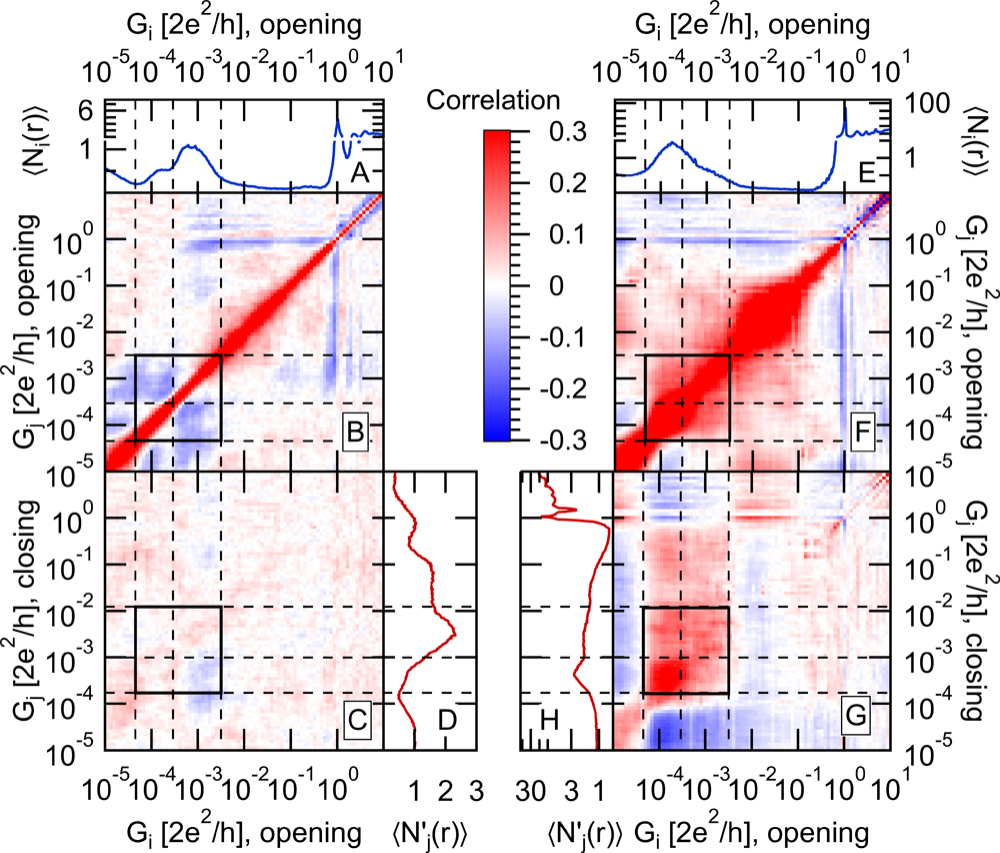
Correlation analysis of the conductance traces. (B, F) Autocorrelation of the room/low-temperature opening traces, calculated as , where *i*, *j*, and *r* are respectively the conductance bin labels and the trace index, the number of data points in bin *i* on the opening trace *r* is *N*_*i*_(*r*), whereas . Note that *G*_*i*_ is the conductance of bin *i*, and  is the one-dimensional conductance histogram of the opening traces, as shown in panels A and E for the room/low-temperature measurements. (C, G) Cross-correlation between the opening and subsequent closing traces. Here, the *C*′_*i*,*j*_ correlation coefficient is calculated by replacing *N*_*j*_(*r*) with *N*′_*j*_(*r*) in the above formula, where *N*′_*j*_(*r*) is the number of data points in bin *j* of the closing trace *r*; i.e., the closing trace right after the opening trace *r*.  is the one-dimensional conductance histogram of the closing traces, as shown on panels D and H for the room/low-temperature measurements. The same color scale is used on all panels to show the value of the correlation functions. The dashed lines indicate the borders of the HighG and LowG molecular configurations both in the opening direction and in the closing direction.

In contrast, the autocorrelation plot of the low-temperature data set (Figure 2F) shows that the conductance regions of the HighG and LowG peaks are positively correlated. We argue that, in this case, the positive correlation originates from the reduced pick-up rate at low temperature. In the room-temperature data set, almost all of the traces are molecular; hence, the correlation plot truly reports about the relation of the two molecular plateaus. The low-temperature data set, however, contains a significant number of tunneling traces as well. The distinction between the tunneling traces (lower than average counts in the molecular region) and the molecular traces (higher than average counts in the molecular region) naturally introduces a positive correlation which suppresses the intrinsic anticorrelation between the two molecular plateaus. Later, we verify this argument by separating the tunneling and the molecular traces and by studying the correlations solely for the latter subset of the traces.

The next question is whether there is any relationship between the junction trajectories during the opening and the subsequent closing of the junction. Such structural memory effects can be investigated with the opening/closing cross-correlation analysis^22,30^ (see the caption of Figure 2 for a brief description). As two extremities, (i) one can consider that the closing traces are completely independent from the previous opening traces due to the significant junction rearrangement after the rupture (see Figure 1B). This case reveals zero opening–closing correlations. (ii) The closing traces may precisely reproduce the previous opening traces; i.e., up to a certain conductance, the closing is exactly the time-reversed process of the opening (see Figure 1A). In the latter case, the opening–closing correlation plot reproduces the opening autocorrelation plot, including the pronounced positive correlation at the diagonal.

In case of the room-temperature measurement, the opening–closing correlation plot (Figure 2C) shows extremely weak correlations compared to the autocorrelation plot (Figure 2B). This supports the hypothesis that, after the rupture, the junction is significantly rearranged, and therefore, the information about the opening contact geometry is lost.

As a very sharp contrast, the low-temperature data set exhibits a very strong rectangular-shaped positively correlated region between the entire molecular conductance ranges of the opening and subsequent closing traces (Figure 2G). Again, we argue that this strong correlation is related to the mixture of molecular and tunneling traces along both the opening and closing directions and to the fact that a molecular (tunneling) opening trace is likely to be followed by a molecular (tunneling) closing trace. This already indicates that, after the rupture of a single-molecule junction, the molecule is not lost from the contact: a molecular junction is also likely to be formed, when the electrodes are closed again. However, for a more detailed investigation of the relation between the opening and subsequent closing molecular conductance plateaus, we again need to filter out the tunneling traces and calculate the correlation matrices solely for the molecular traces.

To separate the molecular traces from the tunneling ones, we used a combined classification method, where the traces with extreme positive or negative principal component projections serve as a training data set for a neural network, which then classifies all traces either to the *tunneling* or to the *molecular* category.^16^ First, this classification was performed according to the tunneling or molecular nature of the opening traces. The 2D opening conductance histograms of these two categories are shown in Figure 3A (tunneling opening traces) and in Figure 3D (molecular opening traces), demonstrating an excellent classification of the two trace classes. The closing 2D histogram for the traces showing tunneling character along the previous opening process also show a very clear tunneling character (Figure 3B). The molecular opening traces, however, are either followed by a molecular or a tunneling closing trace (Figure 3E,G). These two subcategories are separated by an additional classification algorithm according to the tunneling or molecular nature of those *closing traces* that show molecular character along the previous opening trace. In summary, this two-step classification reveals three distinct opening–closing trace pair categories, which are also exemplified by sample trace pairs in Figure 3C,F,H: (i) a molecule is absent during the opening, in this case, the subsequent closing trace also lacks any molecular signatures (≈67% of all traces due to the reduced molecular pick-up rate, Figure 3A–C); (ii) a molecular plateau can be observed both during the opening and the subsequent closing of the junction (≈ 15% of all traces, Figure 3D–F); and (iii) a molecular junction is established during the opening but the molecule is lost after rupturing this contact (≈18% of all traces, Figure 3D,G,H). These results suggest that molecules can only be captured by the electrodes during the opening of the metallic junction. Once the molecular junction is ruptured, the molecule can either stay attached to one of the electrodes and can be recovered during the next closing cycle or it can flip back to the surface of one electrode, yielding only tunneling current during the subsequent closing of the junction.

**Figure 3:**
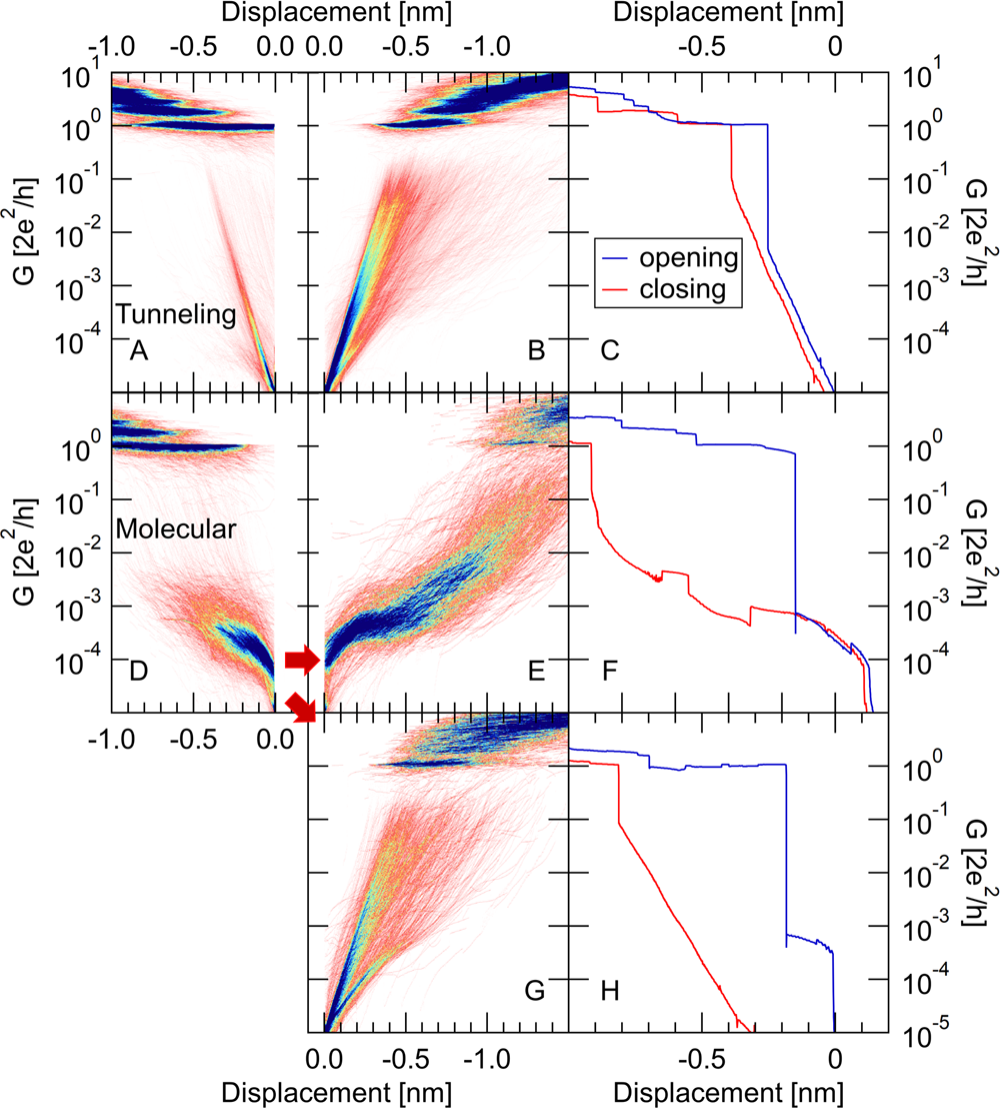
2D opening (A, D) and closing (B, E, G) conductance histograms and sample opening–closing trace pairs (C, F, H) demonstrating the three distinct trace classes of the low-temperature measurements. (A–C) The traces exhibiting tunneling character along the opening of the junction. For these traces, the closing process also exhibits tunneling character (B, C). The rest of the traces exhibit molecular character along the opening of the junction (D). These opening molecular traces are divided to further two categories according to the molecular (E, F) or the tunneling (G, H) character of the subsequent closing traces.

The above analysis shows that ≈45% of the low-temperature molecular opening traces exhibit molecular plateaus along the closing process as well. In the following, we further analyze this subset of the traces (825 traces in the presented data set; see the corresponding opening and closing conductance histograms in Figure 4B,F) and compare their correlation patterns to the room-temperature data, where also both directions exhibit molecular character. As a reference, Figure 4A displays the 2D conductance histograms of this restricted low-temperature data set such that the opening (blue) and closing (red) traces are interlined on the same plot. The gray solid/dashed lines show the average opening/closing traces. This type of visualization already demonstrates that in average, the closing traces exhibit very similar LowG plateaus as the opening ones in a certain displacement range (see the Supporting Information for a more detailed comparison of the opening/closing avarge traces).

**Figure 4:**
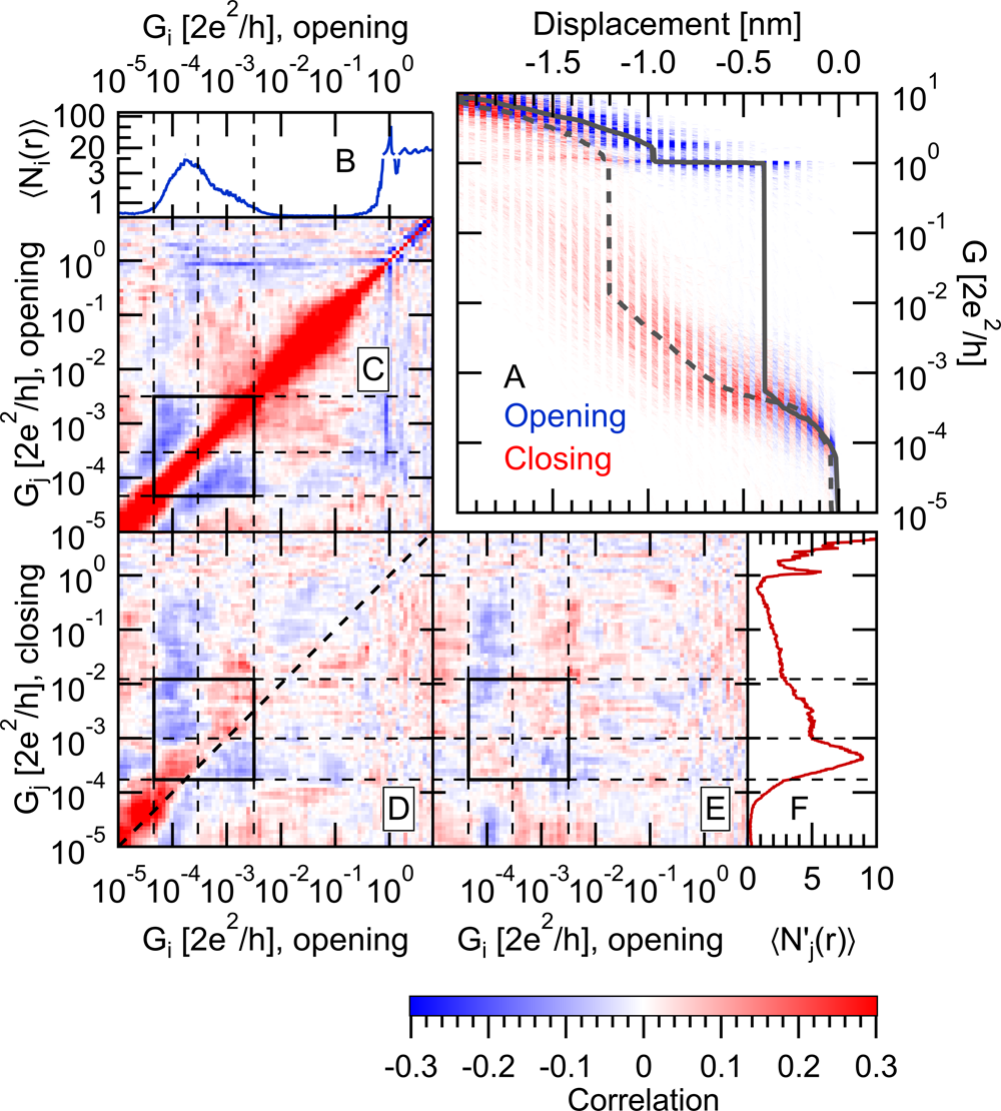
Further analysis of those low-temperature trace pairs, where both the opening and the closing directions exhibit molecular character. (A) Interleaved 2D conductance histograms of the opening (blue) and closing (red) traces. To construct this 2D histogram, the opening–closing trace pairs are aligned by setting the origin of the displacement axis to the point where the opening traces cross the 10^–5^ G_0_ conductance. The average conductance traces are plotted with solid/dashed line in the opening/closing direction. These average traces are extracted by fitting a Gaussian to each column of the two-dimensional histogram and plotting the peak location with respect to the displacement. (C) Autocorrelation plot of the opening traces. (D) Opening–closing cross-correlation plot. As a reference the diagonal and the above-defined borders of the LowG and HighG regions are shown by dashed lines. Panels B and F represent the opening/closing conductance histograms of this restricted data set, whereas panel E is a shifted opening–closing correlation plot, where the opening traces are correlated with the closing traces from the next molecular opening/closing cycle instead of the same opening/closing cycle.

Figure 4C shows the autocorrelation plot for the opening traces of this low-temperature molecular trace class. This autocorrelation plot resembles the room-temperature autocorrelation plot (Figure 2B), in particular, the characteristic anticorrelation of the HighG and LowG molecular plateaus is clearly resolved (see the off-diagonal blocks of the enframed region). Although the relative weights of the HighG and LowG configurations are markedly different in the room- and low-temperature histograms (Figure 1C), the similarity of the autocorrelation plots as well as the HighG and LowG conductance values implies the appearance of similar molecular junction arrangements at both temperatures.

The low-temperature opening–closing cross-correlations (Figure 4D), however, are completely different from the room-temperature data (Figure 2C). The negligible correlations in the latter case are replaced by strong opening–closing correlation features resembling the low-temperature opening autocorrelation plot (Figure 4C) in the low conductance region. In particular, strong positive correlations are observed close to the diagonal, which is a clear indicator that similar, statistically dependent conductance plateaus are formed along the closing traces as along the previous opening traces.^28,29^ Note, however, that this positively correlated region exhibits a slight vertical shift along the closing conductance axis, which we attribute to the elevated conductance of the less stretched closing configurations.

In conclusion, we have compared the room- and low-temperature properties of Au–BP–Au single-molecule junctions. In spite of the markedly different room-temperature and low-temperature weights of the HighG and LowG molecular configurations in the conductance histograms, we have found surprisingly similar patterns in the autocorrelation plots of the opening traces at both temperatures. The latter indicates the formation of similar junction arrangements. On the other hand, the opening–closing cross-correlations exhibit completely different behavior at room/low temperature. At room temperature, the opening–closing correlation vanishes, indicating a significant rearrangement of the molecular junction after its rupture. As a sharp contrast, the low-temperature opening–closing correlation analysis demonstrates the likely scenario that the molecule stays protruding from the apex of one electrode after the rupture, and therefore the very same molecular junction can be recovered as the junction is closed. This property provides some kind of protection against the vulnerability of the single-molecule nanowire due to a temporary disconnection. The absence of this advantageous property in room-temperature measurements is partly related to the intense surface diffusion of gold atoms. However, a proper electrode material choice together with a well-tailored, rigidly bound molecular arrangement may yield similar junction memory features in room-temperature measurements as well.

At this point, it is important to emphasize that opening/closing 2D conductance histograms and average conductance traces (Figure 4A) convey a completely different information than the opening–closing correlation plot (Figure 4D). The 2D histograms show that the opening and closing traces exhibit similar molecular plateaus *on average*. As a very sharp contrast, the opening–closing correlation plot tells us about *the correlated deviation from the average values*. This means that a certain pattern on a particular opening trace (e.g., a longer-than-average LowG plateau and a shorter-than-average HighG plateau) is likely to be reproduced on the subsequent closing trace. This reproducibility is restricted to the molecular conductance range, and to the opening–closing *trace pairs*. Note that the strong correlations vanish above the HighG conductance range (see the vanishing correlations above the conductances of the enframed molecular region in Figure 4D). Furthermore, there are also no strong correlations when the closing traces are shifted, i.e. when examining the relationship between the opening trace in the *r* and the closing trace in the *r* + 1 opening/closing cycle (see the absence of strong correlations around the diagonal in Figure 4E). The above analysis underpins the tendency that is demonstrated by the sample opening–closing low-temperature molecular trace pairs in Figures 1F and 3F. These traces show that not only the presence of the molecule is recovered as the junction is closed but also the patterns of the opening molecular plateaus are reproduced right after the closing. In other words, it is likely to recover the very same molecular configuration with similar response to the junction displacement.

## Supporting Information Available

Demonstration of the two-dimensional conductance vs displacement histograms and discussion of the conductance difference between the opening and closing molecular configurations (PDF)
