# Supplementary material for: Structural Memory Effects in Gold–4,4′-Bipyridine–Gold Single-Molecule Nanowires
Source: J Phys Chem Lett. 2021 Feb 11;12(7):1759–64. doi: 10.1021/acs.jpclett.0c03765 (PMC8023710; doi:10.1021/acs.jpclett.0c03765)
Supplement: Supplementary file 1 — jz0c03765_si_001.pdf [file jz0c03765_si_001.pdf]

Supporting Information
Structural Memory Effects in Gold–4,4'-Bipyridine–Gold Single-Molecule Nanowires

A. Magyarkuti,† Z. Balogh,*,†,‡ G. Mezei,†,‡ and A. Halbritter†,‡

†*Department of Physics, Budapest University of Technology and Economics, Budafoki ut 8, 1111 Budapest, Hungary*

‡*MTA-BME Condensed Matter Research Group, Budafoki ut 8, 1111 Budapest, Hungary*

E-mail: balogh.zoltan@mail.bme.hu

Two-dimensional histograms

Supporting Figure 1 shows the two-dimensional conductance vs. displacement histograms[1,2] of room temperature (A,B) and low temperature (C,D) Au–4,4' bipyridine–Au single-molecule nanowires both in the opening (A,C) and closing (B,D) direction. These 2D histograms confirm, that molecular plateaus extend to significantly longer displacements during the closing of the junction compared to the opening direction. Furthermore, these two-dimensional histograms reveal, that at low temperature a significant number of traces are measured without molecular signatures. This is not evident from the one-dimensional conductance histograms, since tunneling traces do not produce peaks. However, when the traces are aligned at a conductance value just above the noise limit of the measurement, tunneling traces overlay onto a single exponential curve due to their

well-defined displacement characteristics. Such features can be observed on the two-dimensional histograms of the low-temperature measurement (Supporting Figure 1/C,D), indicating that tunneling traces are mixed with molecular ones in both the opening and the closing direction. In contrast, the two-dimensional histograms of the room-temperature measurement do not indicate significant number of tunneling traces in either direction, thus almost all opening and closing conductance traces exhibit molecular plateaus (Supporting Figure 1/A,B).

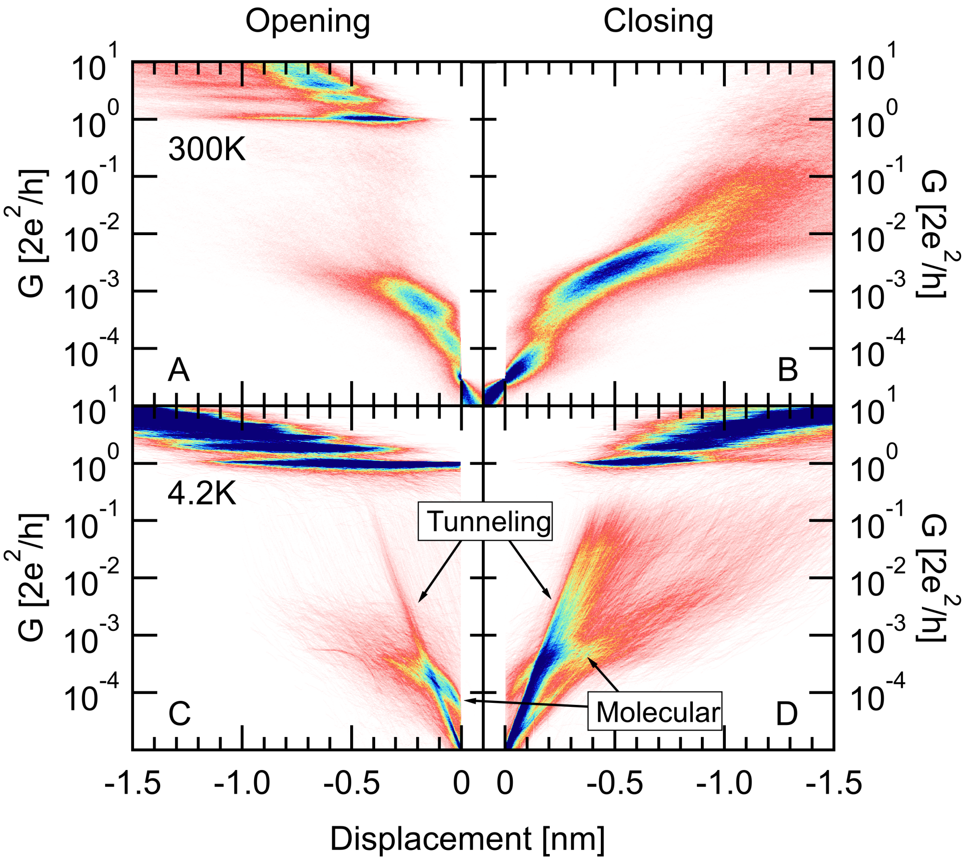

Figure 1: *Two-dimensional conductance vs. displacement histograms of gold–4,4' bipyridine–gold single-molecule nanowires at room temperature (A,B) and $T = 4.2\,K$ (C,D). Panels (A,C) represent the 2D histograms of the opening traces, whereas the 2D histograms of the closing traces are plotted in (B,D). In case of the low temperature measurements molecular traces are mixed with tunneling traces (see the arrows).*

Discussion of the conductance difference between the opening and closing molecular configurations

In the manuscript both the room temperature and low temperature conductance histograms demonstrate a significant conductance difference between the opening and closing molecular configurations (See Fig. 1/C,D in the manuscript). Here, we further discuss this difference by re-plotting the low temperature average conductance traces from Fig. 4/A in a magnified way (see Supporting Figure 2/A), and comparing these average traces by the opening and closing conductance histograms of the same restricted dataset (see Supporting Figure 2/B). The similarity of the opening and closing LowG conductance plateaus is restricted to a certain displacement range with clear differences at the beginning and end of the plateaus. On one hand ≈ 0.3 Ångstrom hysteresis is observed around the rupture point, i.e. the final configuration is a bit stretched before the rupture, and the jump back to contact happens at a bit smaller electrode separation. This difference yields a slight offset between the low-conductance tails of the opening and closing conductance histograms (see Supporting Figure 2/B). More importantly, the average closing trace exhibits an extended LowG plateau around 3 Ångstrom reverse displacement from the rupture point on the opening trace. Naturally, the conductance of this plateau is aligned with the position of the LowG peak in the closing histogram (see the red dotted line in the Supporting Figure 2/B). However, on the opening trace the major part of this plateau is not sampled, as the jump from the gold single-atom junctions or atomic chains to the LowG configuration happens at a displacement range, where only the final part of this plateau is observed. This difference yields a significantly lower peak position and a significantly suppressed peak amplitude in the opening conductance histogram compared to the closing histogram. These two differences between the opening and closing traces are also exemplified the the opening-closing trace pair in Fig. 3/F of the manuscript.

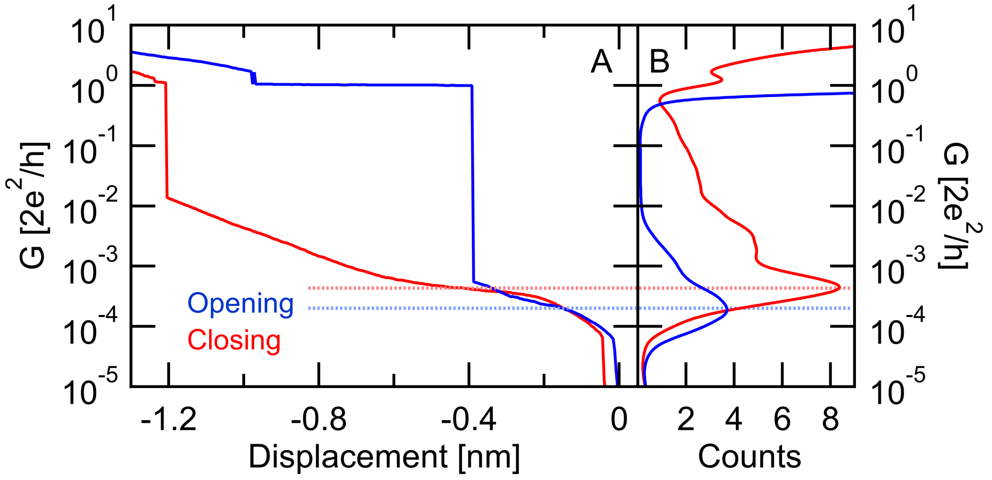

Figure 2: *(A) Average opening (blue) and closing (red) conductance traces reproduced from Fig. 4/A with better visibility. To identify the relevant conductance regions the opening (blue) and closing (red) conductance histograms are also reproduced from Fig. 4/B,F in a smoothed fashion, and the peak positions are projected to the average traces (blue and red dotted lines).*

References

(1) Martin, C. A.; Ding, D.; Sørensen, J. K.; Bjørnholm, T.; Van Ruitenbeek, J. M.; Van Der Zant, H. S. Fullerene-Based Anchoring Groups for Molecular Electronics. *Journal of the American Chemical Society* **2008**, *130*, 13198–13199.

(2) Quek, S. Y.; Kamenetska, M.; Steigerwald, M. L.; Choi, H. J.; Louie, S. G.; Hybertsen, M. S.; Neaton, J. B.; Venkataraman, L. Mechanically Controlled Binary Conductance Switching of a Single-Molecule Junction. *Nature Nanotechnology* **2009**, *4*, 230–234.
